# The influence of pole lengths on O_2_-cost, kinematics, and performance in double poling at high speeds before and after a training period with long poles

**DOI:** 10.1007/s00421-019-04237-z

**Published:** 2019-09-30

**Authors:** Thomas Losnegard, Ola Kristoffer Tosterud, Erik Trøen, Camilla Høivik Carlsen, Gøran Paulsen, Bjarne Rud

**Affiliations:** 1 Department of Physical Performance, The Norwegian School of Sport Sciences, Ullevål Stadion, Post box 4014, 0806 Oslo, Norway; 2 The Norwegian Olympic and Paralympic Committee and Confederation of Sports, Oslo, Norway

**Keywords:** Cross-country skiing, Cross-country skiers, Economy, Equipment, Center of mass, Skiing technique

## Abstract

**Purpose:**

Previous studies have found an acute performance improvement with longer pole lengths in double poling (DP) at low-to-moderate speeds. We investigated the influence of pole lengths (PL) on O_2_-cost, 3D kinematics, and performance in DP at moderate-to-high speeds before (Pre) and after (Post) eight training sessions with long poles on a rollerski treadmill.

**Methods:**

Seven male and four female skiers completed tests with two different PLs (84 and 90% of body height). Submaximal O_2_-cost (1º; 4.5 [females] or 6 m s^−1^ [males]) and a peak velocity test (1º; ∼  7.3 m s^−1^) were assessed before and after a six week training period. The training sessions consisted of 50 min of low-moderate intensity training and 4 × 10 s maximal sprints with PL^90%^.

**Results:**

On average for all tests, PL^84%^ induced 1.0 ± 1.0% higher peak velocity compared to PL^90%^ (mean ± CI) with no difference in vertical displacement of center of mass (COM_z_). From Pre to Post, peak velocity and cycle time were increased and the displacement of COM_z_ were reduced similarly for both PLs. At moderate speed, PL^90%^ induced less displacement of COM_z_ with subsequent 1.1 ± 0.7% lower O_2_-cost compared to PL^84%^. From Pre to Post, the O_2_-cost and COM_z_ were reduced similarly for both PLs.

**Conclusions:**

Longer PL than skiers self-selected lengths reduce O_2_-cost at moderate speeds, but induced lower peak velocity. Eight sessions of training with PL90% did not influence the difference between PL^84%^ and PL^90%^ on O_2_-cost, kinematics or peak velocity.

## Introduction

Classical cross-country skiing consists of three main techniques: Diagonal stride, double poling with kick and double poling (DP). The choice of these sub-techniques depends mainly on speed, and therefore, they act as a gearing system. The “high speed” DP technique has evolved over the last decade, given the nature of today’s races with substantially higher average speeds (Losnegard 2019). Furthermore, as 5 out of 6 events in the Olympics today are mass starts, the outcome is often decided by the ability to generate high speeds during the race and/or in the end-spurt to break away from the group. Accordingly, enhancing DP performance has gained increased attention from a physiological, technical and equipment perspective (Stöggl and Holmberg 2016; Børve et al. 2017; Carlsen et al. 2018; Stöggl and Karlöf 2013; Losnegard et al. 2017).

During DP, propulsive forces are transferred through the poles, suggesting that pole length (PL) is an important parameter in determining O_2_-cost and performance (Carlsen et al. 2018; Losnegard et al. 2017; Hansen and Losnegard 2010; Onasch et al. 2017). The consensus of previous studies is that PLs up to ~ 90% of actual body height reduce the vertical displacement of the center of mass (COM_z_), which leads to a lower O_2_-cost and increased performance compared to PLs of ~ 84% of body height (Carlsen et al. 2018; Losnegard et al. 2017; Onasch et al. 2017). However, with the exceptions of Hansen and Losnegard (2010), studies have exclusively investigated the influence of PL in DP at low-to-moderate skiing speeds (< 5.5 m s^−1^) (Hoffman et al. 1994; Carlsen et al. 2018; Losnegard et al. 2017; Nilsson et al. 2003; Onasch et al. 2017). Thus, little knowledge exists about how PL influences O_2_-cost, performance and kinematics at higher speeds. Moreover, in all previous studies, only the acute effect of changing PL was addressed. Since PL induces clear alterations in skiers’ movement patterns (Carlsen et al. 2018; Losnegard et al. 2017), it has been proposed that familiarization with longer poles is necessary to optimize the coordination of movements and thus performance (Hansen and Losnegard 2010). This is of special importance at high speeds, where the timing of force applications is a crucial determinant of DP performance (Stöggl and Holmberg 2011). However, how cross-country skiers adapt to change in equipment in general, and PL more specifically, has not previously been determined.

The self-selected PL in classical cross-country skiing has traditionally been 82–85% of body height (Hansen and Losnegard 2010). However, over the last decade, several skiers have experimented with longer PLs during training and competitions. Consequently, from 2016 to 2017 season, a temporary rule from the International Ski Federation (FIS) has restricted the classical PL to 83% (including ski boots, equivalent to ~ 85% of actual body height) (FIS 2017). FIS stated that this was to “protect classical technique and all its aspects” and “so that competitions in classical technique are fair for everybody” (FIS 2017). Therefore, since longer PLs enhance performance or performance-related factors during DP in uphill’s compared to self-selected PL (Carlsen et al. 2018; Losnegard et al. 2017), the intention from FIS was that skiers should use less DP during uphill’s and more of other skiing techniques such as the diagonal stride. However, during an international race course consisting of 1/3 uphill, 1/3 flat and 1/3 downhill (Losnegard 2019), the rational for elite skiers using longer PL in uphill’s clearly demands that performance is not affected negatively in flat terrains at higher speeds. To date, scientific evidences on the effect of PL in these sections on performance are still limited.

The purpose of this study was twofold. First, we investigated the influence of PLs in DP on O_2_-cost, kinematics and performance at moderate-to-high speeds. Second, the influence of PL was assessed after eight training sessions with longer poles, conducted in controlled conditions on a rollerski treadmill. We hypothesized that a longer PL (90% of body height) would reduce O_2_-cost, COM_z_ and increase peak speed compared to a PL of 84%. Moreover, we hypothesized that the differences between PLs would increase after training with long poles, due to enhanced technique in DP at high speeds.

## Methods

### Subjects

Seven male and four female cross-country skiers (mean ± SD; 20 ± 3 years; 176 ± 11 cm; 69 ± 8 kg) participated in the study. An inclusion criterion was competing in the Norwegian national cup for seniors or placing in the top 30 in the Norwegian cup for juniors. Participants’ self-selected classic style PL was 147 ± 9 cm (84 ± 1% of body height). Their maximal oxygen uptake, tested during treadmill running on a separate day, was 70 ± 8 mL kg^−1^ min^−1^ (range 57–80 mL kg^−1^ min^−1^). For protocols, see Losnegard et al. (2014). The study was conducted according to the Declaration of Helsinki and Norwegian law. All the subjects gave their written informed consent before study participation.

### Experimental design

All tests and trainings in the study were performed on a rollerski treadmill using DP. Prior to the study, the subjects were familiar with treadmill skiing from earlier training and testing. The subjects met on 2 days (called Pre1 and Pre2) within 10 days before and 1 day after the training intervention (called Post). Pre1 was identical to the test procedure conducted at Pre2 and Post as described below, with the exception of capturing 3D kinematics.

The PL was chosen based on a previous study, where skiers using PL^90%^ reduced O_2_-cost more than with PLs of 87, 84, and 82% of body height (Carlsen et al. 2018). The order of PLs was counter-balanced between subjects, but was identical for each subject in all tests. Based on pilot testing, the incline (1°) and speeds were chosen to induce a relevant DP technique in “flat terrain” and to obtain steady-state oxygen uptake. Before and after the training intervention, subjects performed submaximal tests and a peak velocity test including 3D kinematics with both pole lengths. The training intervention lasted 6 weeks (October–November) and consisted of eight training sessions of 50 min DP with PL^90%^ at low-to-moderate intensity and 4 × 10 s sprint at the individual skier’s peak velocity.

### Protocol and measurements

#### Submaximal workload and peak velocity tests

All tests were conducted at 1° inclination. Subjects performed a 10 min warm-up (3–4 m s^−1^, females and males, respectively) and then completed 5 min of roller skiing at submaximal conditions at 1° of 4.5 m s^−1^ (females) or 6.0 m s^−1^ (males). The O_2_-cost was determined as the average oxygen uptake (ml kg^−1^ min^−1^) from 2.5 to 4.5 min and heart rate (HR) was averaged over the same timeperiod. Because of the O_2_ measurement, the subjects were unable to express their rating of perceived exertion (RPE) during the trial. Therefore, at 4 min into the trial, subjects were asked to choose their RPE (6–20), which they then reported at the end of the trial. After a 10 min active recovery (3–4 m s^−1^), skiers performed a peak velocity test separated by 10 min active recovery between PLs. The peak velocity test started at 4 or 5 m s^−1^ (female and males, respectively) and the speed was increased by 0.25 m s^−1^ every 10th second. Participants were instructed to position their front wheels between two laser beams (60 cm apart). If subjects passed the rear laser with their front wheels, the test was abandoned. The highest speed conducted over 10 s was considered peak velocity.

#### 3D kinematics

The 3D kinematics of the body, poles, and rollerskis were collected within the last 15 s during the submaximal tests (average over five cycles analyzed, sampling rate 300 Hz). Fifteen seconds before recording, the mouthpiece and sampling tube for the O_2_ measurements were removed without stopping the treadmill (to avoid reflection from the tube). During the performance test, recording was started 30 s before the time at which subjects reached the highest speed obtained during Pre1 (6.8 ± 0.7 m s^−1^). The highest individual speeds reached at both Pre2 and Post (average speed; 7.3 ± 0.7 m s^−1^) were used for further kinematical analyses (average over five cycles analyzed during the 10 s with sampling rate 300 Hz).

Prior to each session, the motion capture system was calibrated following the manufacturer’s guidelines. Anthropometrical measurements of each subject (body height, length of leg, thorax, head plus neck and circumference of chest, right upper arm (proximal), elbow, wrist, thigh (proximal), knee joint, and ankle joint) were acquired as described in detail previously (Carlsen et al. 2018). For the construction of the modified 3D kinematic model, 41 reflective markers (spherical, 7 mm) were attached over bony anatomical landmarks (pelvis, thorax, and right upper and lower extremities). In addition, two markers were placed on each pole (lateral aspect), 10 cm and 100 cm from the grip; two markers were placed on each roller ski, in front of the rear wheel and behind the front wheel; and two markers were placed on the treadmill (85 m apart) parallel to the skiing direction.

#### Training intervention

A total of eight training sessions were conducted over a period of 6 weeks. Participants engaged in one session per week for the first 4 weeks, and two sessions per week for the last 2 weeks. Each session consisted of 50 min of continual DP divided into 5 × 10 min bouts. Each bout had three different conditions: 5 min at 1º, 3 min at 2º, or 3º and 2 min at 4º or 5º (females and males, respectively). The different conditions were matched for similar external workloads and were individually set (70–80% of maximal heart rate) based on the levels of the skiers. Ninety seconds after the 50 min session, subjects conducted four sprint trials with 90 s break (passive recovery). Subjects started at 5.0 m s^−1^ for 10 s, and then, the speed was increased to the highest speed performed during the last session. When subjects were able to complete all four trials within the laser zone, the speed was increased by 0.25 m s^−1^ for the next session. Seven skiers completed all eight training sessions, two subjects performed seven sessions, and two subjects performed six sessions.

#### Apparatus

All tests were performed on a 3 × 4.5 m treadmill (Rodby, Södertalje, Sweden). Prior to, during and after the testing period, inclines and speed were controlled. All subjects used the same pair of rollerskis (Swenor Fibreglass, Swenor, Sarpsborg, Norway) with wheel types 2 (front) and 3 (rear) and an NNN-binding system (Rottefella, Klokkarstua, Norway). The rollerskis had a friction coefficient of *µ* = 0.023, which did not change during the testing period. The subjects used Swix Triac 1.0 poles (Swix, Lillehammer, Norway) with a tip customized for treadmill rollerskiing. Before the tests, the tips were adjusted to provide identical grip and weight.

Oxygen consumption was measured using an automatic ergospirometry system (Oxycon Pro, Jaeger GmbH, Hoechberg, Germany), as evaluated by Foss and Hallen (2005). Heart rate was measured with a Polar V800 (Polar Electro OY, Kempele, Finland). Anthropometrics were measured with a stadiometer (Seca 213, Hamburg, Germany) and measuring tape. Body mass (net mass and with equipment) was measured using a Seca scale (model 708, Hamburg, Germany).

Kinematic data were collected using a 3D motion capture system (ProReflex, Qualisys, Sävedalen, Sweden) with Qualisys Track Manager software (QTM) 2.7 and 14 cameras (Oqus 4, Qualisys Medical AM, Göteborg, Sweden). The global coordinate system was defined as follows: the incline of the treadmill was set to 0˚; the *X*-axis was the longitudinal axis of the treadmill (the direction of motion); the *Y*-axis was the side-to-side direction across the treadmill; and the *Z*-axis was perpendicular to the ground. Visual 3D (C-motion, Inc., USA) and MATLAB (MathWorks, Inc., Natick, MA, USA) were used for further analysis.

#### Data analysis

Kinematic raw data were filtered (fourth-order Butterworth low-pass filter, cut-off frequency of 6 Hz) and further processed in Visual3D and MATLAB. Cycle time was defined as the time between two pole plants, poling time as the time between pole plant and subsequent pole lift-off, and reposition time as the time between pole lift-off and subsequent pole plant. Pole plant and pole lift-off were determined from the path of the pole markers in Visual3D, where pole plant was determined as the maximum forward position in the horizontal plane and pole lift-off was determined as the minimum vertical value in the sagittal plane. The pole angles relative to the treadmill belt plane at pole plant and pole lift-off were calculated in Visual3D.

The COM_z_ was derived from seven body segments (forearm including the hand, upper arm, trunk and head, pelvis, thigh, leg, and foot), together with two segments for ski and pole. The relative mass of each body segment with respect to the total body mass was calculated based on de Leva (1996). The equipment was weighed independently, and the weights of the ski boots were added to the foot segment. Each body segment’s COM was calculated with respect to its proximal segmental reference (de Leva 1996), and the COM for the whole body plus equipment was calculated.

Pole angles, cycle characteristics, and COM_z_ were calculated from five consecutive cycles. For comparison, each cycle was time-normalized using a third-order 101 point interpolation. Pole angles and the vertical position of COM_z_ for each pole length were compared at pole plant, pole lift-off, maximum (max) and minimum (min) values during the cycle. The displacement of COM_z_ was calculated from the maximal and minimum values of the position of COM_z_ (COM_zmax_–COM_zmin_), regardless of when in the cycle it appeared. The shortest distances between COM and the poles at pole plant (D_COM-pole plant_) were calculated from COM_z_ and the coordinates of the pole markers. Due to missing data, D_COM-pole plant_ calculations were only conducted for the submaximal tests.

### Statistical analysis

Normality of the data was assessed using the Shapiro–Wilks test of normality (*a* = 0.05). Raw data are presented as mean ± standard deviation (SD) and RPE is presented as median ± interquartile range (IQR). Relative differences between pole lengths are presented as mean ± 95% confidence interval (CI). The differences between two different pole lengths at three test periods were conducted (two at pretests: Pre1, Pre2 and one at posttest; Post). Initially, the main effects of pole length (PL^84%^ and PL^90%^) and time, as well as their interaction, were analyzed with a two-factor within-subject ANOVA (2 × 3 design for O_2_-cost and peak velocity: Pre1, Pre2, and Post). 3D kinematics, HR, and RPE were only analyzed at Pre2 and Post. Therefore, a 2 × 2 design (Pre2 and Post-test) for these variables was conducted. If a main effect of pole length was found, a one-way ANOVA comparing pole lengths for each of the time periods was conducted separately, followed by a Tukey’s post-hoc correction for multiple comparisons. Where sphericity assumption was violated, *P *values were adjusted according to Geisser–Greenhouse. Statistical calculations were performed using Microsoft Office Excel 2013 (Microsoft, Redmond, USA), SigmaPlot version 13.0 software (San Jose, CA, USA, and Graph Pad prism 8.2 (San Diego, CA, USA). The level of confidence was set to 95% and a *P *value ≤ 0.05 was considered statistically significant.

## Results

### Peak velocity

**Fig. 1:**
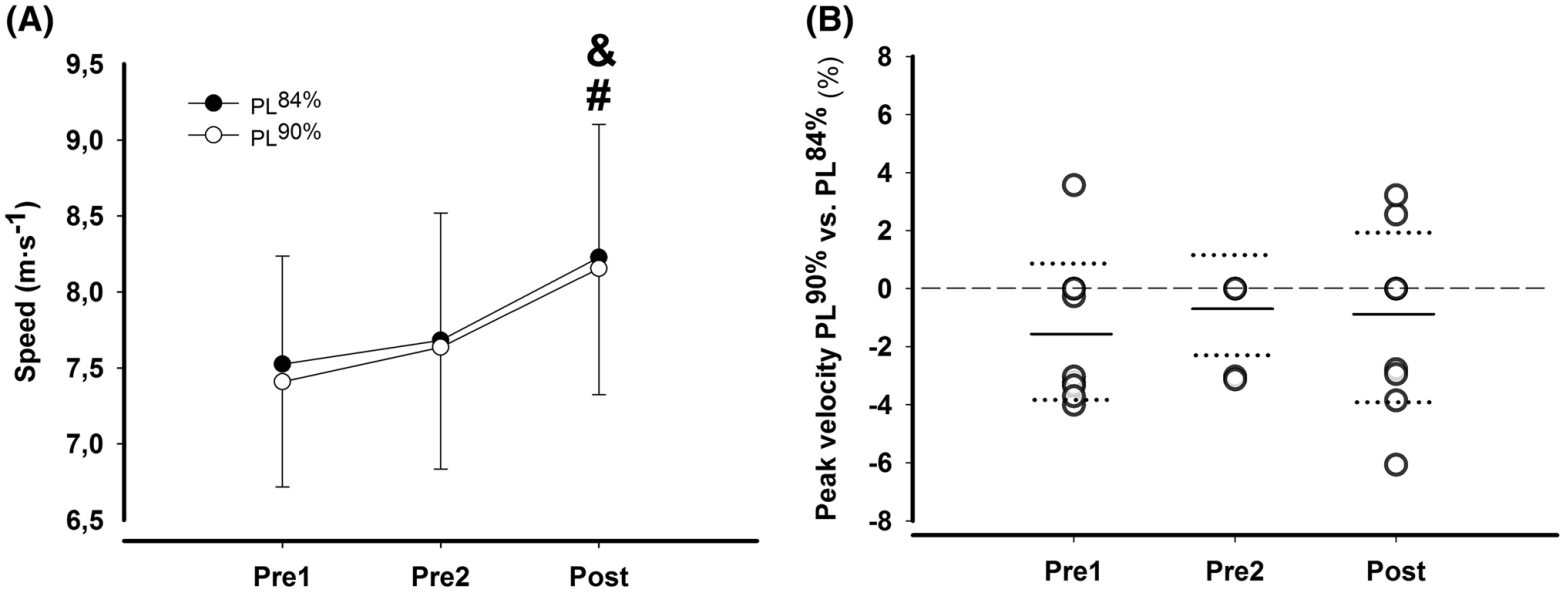
**a** Peak velocity at Pre1, Pre2, and Post for PLs of 84 and 90% of body height. Data are presented as mean ± SD. **b** Individual differences (%) in peak velocity at Pre1, Pre2, and Post between PL of 84 and 90% of body height. Horizontal full lines indicate mean differences, and horizontal dotted lines show upper and lower 95% CI. ^&^Difference to Pre2, ^#^difference to Pre1 (*P* < 0.05)

There was a significant main effect of pole length [*F*(1, 10) = 10.8, mean ± CI; 1.0 ± 1.0%, *P* = 0.05] and time [*F*(1.5, 15) = 44.6, *P* < 0.001], but there were no significant interactions between time and pole length [*F*(1.7, 17.4) = 0.5, *P* = 0.60]. No significant differences were found between pole lengths in peak velocity at Pre1 (1.5 ± 2.4%, *P* = 0.30), at Pre2 (0.6 ± 1.7%, *P* = 0.82) or Post (− 0.9 ± 2.8%, *P* = 0.86) (Fig. 1a, b).

Peak velocity did not increase significantly from Pre1 to Pre2 (2.1 ± 3.1%, *P* = 0.27 and 3.1 ± 3.7%, *P* = 0.12), but increased significantly from Pre1 to Post (9.3 ± 4.6%, and 10.0 ± 5.0%, and Pre2 to Post (7.1 ± 3.6%, and 6.8 ± 3.9%1), (all *P* < 0.001), PL^84%^ and PL^90%^, respectively.

Temporal patterns and kinematics are presented in Table 1 and Fig. 3. For cycle time, poling time, and reposition time, there was no significant main effect of pole length [*F*(1, 10) = 0.8–2.0, *P* = 0.19–0.40], a significant main effect of time [*F*(1, 10) = 8.6–13.2, *P* < 0.01], and no interaction [*F*(1, 10) = 0.6–1.4, *P* = 0.27–0.44]. For the displacement of COM_z_, there was no significant main effect of pole length [*F*(1, 9) = 0.28, *P* = 0.61], a significant main effect of time [*F*(1, 9) = 18.5, *P* = 0.002], and no interaction [*F*(1, 9) = 0.08, *P* = 0.79]. For pole angle at pole plant, there was a significant main effect of pole length [*F*(1, 10) = 5.5, *P* = 0.04], a significant main effect of time [*F*(1, 10) = 26.2, *P* = 0.001], but no interaction [*F*(1, 10) = 0.5, *P* = 0.53]. For pole angle at pole leave, there was a significant main effect of pole length [*F*(1, 10) = 4.8, *P* = 0.05], a significant main effect of time [*F*(1, 10) = 11.4, *P* = 0.007], but no interaction [*F*(1, 10) = 0.2, *P* = 0.65].

**Table 1:** Cycle time, poling time, reposition time and vertical displacement of center of mass (COM_z_) at submaximal workload and at peak velocity test

	Pre2		Post	
	PL^84%^	PL^90%^	PL^84%^	PL^90%^
**Submaximal workload**				
Cycle time (s)	1.20 ± 0.07	1.22 ± 0.09	1.28 ± 0.08	1.29 ± 0.14
Poling time (s)	0.35 ± 0.04	0.36 ± 0.05	0.38 ± 0.04	0.39 ± 0.04
Reposition time (s)	0.85 ± 0.07	0.85 ± 0.07	0.90 ± 0.07	0.90 ± 0.08
COM_z_ displacement (cm)	19.8 ± 3.5	18.5 ± 3.8	17.5 ± 2.4^a^	16.9 ± 2.4
Pole angle_pole plant_ (˚)	87 ± 4	87 ± 5	88 ± 48	87 ± 4
Pole angle_pole lift-off_ (˚)	28 ± 2	28 ± 3	28 ± 2	27 ± 2
D_COM_-pole plant (cm)	51.1 ± 5.0	49.0 ± 5.7^b^	51.2 ± 5.7	48.4 ± 6.2^b^
**Peak velocity**				
Cycle time (s)	0.89 ± 0.12	0.86 ± 0.12	1.02 ± 0.11^a^	1.02 ± 0.13^a^
Poling time (s)	0.24 ± 0.02	0.24 ± 0.02	0.28 ± 0.03^a^	0.28 ± 0.03^a^
Reposition time (s)	0.65 ± 0.12	0.62 ± 0.11	0.74 ± 0.09	0.74 ± 0.11
COM_z_ displacement (cm)	26.2 ± 3.4	25.8 ± 3.8	23.6 ± 2.7^a^	23.5 ± 3.0
Pole angle_pole plant_ (˚)	80 ± 5	78 ± 4	85 ± 6^a^	84 ± 5^a^
Pole angle_pole lift-off_ (˚)	23 ± 2	23 ± 1	24 ± 1	24 ± 2

Speed was set identical within participants between tests for submaximal workload and peak velocity test

Data are mean ± SD

D_COM_-pole plant (cm) is the distance between center of mass and pole at pole plant

^a^Significant difference between Pre2- and Posttest

^b^Significant between pole lengths

### Submaximal workload

**Fig. 2:**
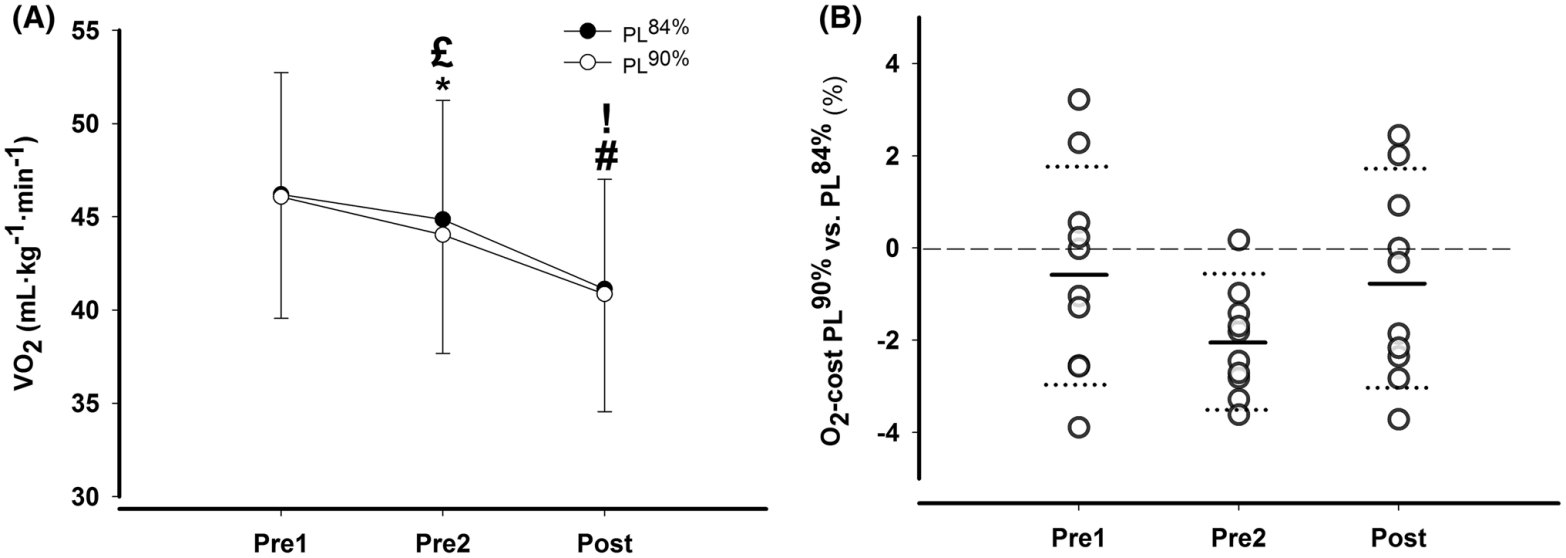
**a** O_2_-cost at Pre1, Pre2, and Post for PLs of 84 and 90% of body height. Data are presented as mean ± SD. **b** Individual difference (%) in O_2_-cost at Pre1, Pre2, and Post between PLs of 90 and 84% of body height. Horizontal full lines indicate mean differences, and horizontal dotted lines show the upper and lower 95% CI. *Difference between pole lengths, ^#^difference to Pre1, ^£^different to Pre1 for PL of 90% of body height, ^!^different to Pre2 for PL of 84% of body height (*P* < 0.05)

On O_2_-cost, it was a significant main effect of pole length [*F*(1, 9) = 11.5, 1.1 ± 0.7%, *P* = 0.008] and time [*F*(1.3, 12.1) = 17.9, *P* < 0.001], but with no interaction [*F*(1.8, 16.4) = 1.9, *P* = 0.18]. Post-hoc tests revealed no difference in O_2_-cost between PLPL^90%^ and ^84%^ at Pre1 (− 0.6 ± 2.4%, *P* = 0.94). At Pre2, PL^90%^ induced a 2.1 ± 1.5% lower O_2_-cost compared to PL^84%^ (*P* = 0.007), while at Post, no difference was found (− 0.7 ± 2.4%, *P* = 0.92). O_2_-cost did not change significantly from Pre1 to Pre2 for PL^84%^ (3.0 ± 4.3%, *P* = 0.24), but decreased 4.4 ± 4.4% for PL^90%^ (*P* = 0.05). From Pre2 to Post, O_2_-cost decreased for PL^84%^ (6.9 ± 5.7%, *P* = 0.02) and tended to decrease for PL^90%^ (5.6 ± 5.8%, *P* = 0.07). From Pre1 to Post, O_2_-cost decreased for PL^84%^ (9.7 ± 7.5%, *P* = 0.012) and for PL^90%^ (9.8 ± 7.7%, *P* = 0.014) (Fig. 2a, b).

For heart rate, there was no significant main effect of pole length [*F*(1, 10) = 0.33, *P* = 0.57], a significant main effect of time [*F*(1, 10) = 18.7, *P* = 0.002], and no interaction [*F*(1, 10) = 1.8, *P* = 0.20]. Heart rate were reduced from Pre1 to Post by 5 ± 3% for PL^84%^ (*P* = 0.006) and 4 ± 3% for PL^90%^ (*P* = 0.013). For RPE, no difference was found between PL at Pre2 or Post and no changes from Pre2 to Post for PL^84%^ (median ± IQR) (14 ± 2 and 14 ± 1), or PL^90%^ (14 ± 1 and 14 ± 1).

**Fig. 3:**
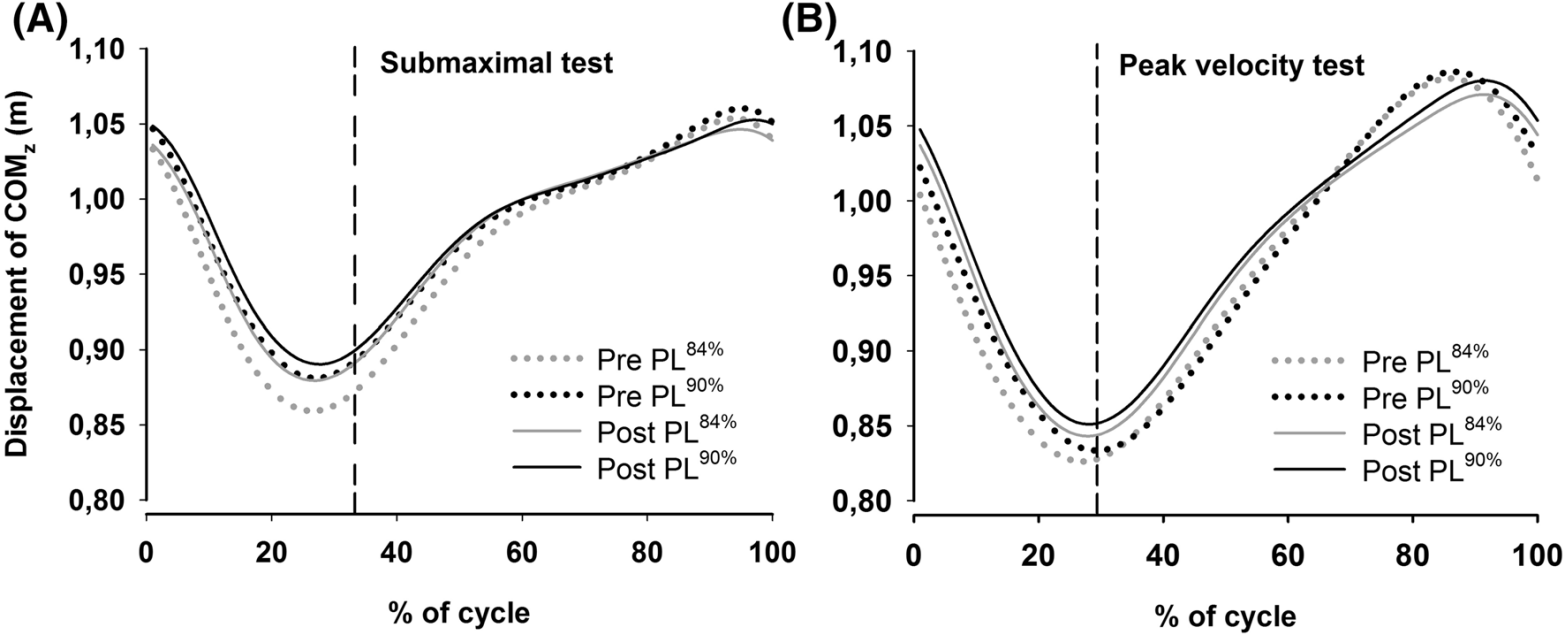
Vertical displacement of center of mass (COM_z_) during a full cycle at submaximal test (4.5 [females] or 6 m s^−1^ [males]) and at peak velocity test (7.3 ± 0.7 m s^−1^) at Pre2 and Post for PL^84%^ and PL^90%^. Each curve represents an average of five cycles for each subject. The cycle starts (0%) and ends (100%) at pole plant. Vertical dotted line indicates pole lift-off

Temporal patterns and kinematics are presented in Table 1. No significant differences were found in cycle time, poling time, or reposition time between pole lengths at Pre2 or Post. Displacement of COM_z_ during a full cycle is shown in Fig. 3. It was a significant main effect of pole length [*F*(1, 9) = 22.9, *P* = 0.001], a significant main effect of time [*F*(1, 9) = 11.6, *P* = 0.008], but no interaction [*F*(1, 9) = 0.55, *P* = 0.48]. Post-hoc tests revealed a significant reduction in displacement of COM_z_ from Pre2 to Post-test only for PL^84%^. For the distance between the COM and the pole (*D*COM-pole plant), it was a significant main effect of pole length [*F*(1, 10) = 26.1, *P* < 0.001], but not with time [*F*(1, 10) = 0.04, *P* = 0.84] and no interaction [*F*(1, 10) = 0.93, *P* = 0.36] (Table 1). No differences were found in pole angle at pole plant or pole leave at Pre2 or Post for either pole length.

## Discussion

This study investigated the influence of two different pole lengths (84 and 90% of body height) on O_2_-costs, kinematics, and peak velocity during treadmill DP before and after eight training sessions using PL^90%^. The principal findings were: (1) use of PL^90%^ reduced the displacement of COM_z_ and O_2_-cost compared to PL^84%^ at moderate speeds. However, at high speeds, PL^84%^ resulted in a higher peak velocity compared to PL^90%^. (2) Eight training sessions with PL^90%^ increased peak velocity and reduced the O_2_-costs and displacement of COM_z_ with both pole lengths. However, the differences between pole lengths found at pretests in O_2_-cost, displacement of COM_z,_ and peak velocity did not change after the training period.

### Effects of pole lengths at moderate-to-high speeds

Although the influence of pole length in DP has been widely studied over the last decade, few studies have investigated how pole lengths influence performance and performance-related factors at high speeds. Hansen and Losnegard (2010) investigated the effect of three different PLs (~ 80, 84, and 88% of body height) on 80-m sprint performance on snow and found that PL^88%^ was faster than PL^84%^ and PL^80%^. However, the benefits of PL^88%^ were most pronounced in the acceleration phase, where the speed was low to moderate (< 5 m s^−1^). Moreover, in all the previous studies that have found a significant effect of longer poles, the speed was low to moderate (< 5.5 m s^−1^), with correspondingly greater inclination (1.7–4.5°) (Losnegard et al. 2017; Carlsen et al. 2018; Onasch et al. 2017). The present study showed that using pole lengths of PL^84%^ increased peak velocity compared to PL^90%^ in DP in “flat terrain” (1º) during treadmill rollerskiing. This implies that speed and incline are dependent factors for the performance effects of pole lengths in DP. This involves at least two key factors: First, as stated in the previous work, DP in different terrains and speeds demands different movement patterns to overcome the external force (Carlsen et al. 2018; Stöggl and Holmberg 2016). In uphill slopes, work against gravity and the ability to use the lowered COM, potentially with a small distance between COM and poles at pole plant, has previously been related to the main mechanism behind reduced O_2_-cost and enhanced performance with longer poles in DP (Carlsen et al. 2018). However, in flat terrain and at high skiing speeds, an optimal timing of pole force over a short poling time is the primary limiting factor. As speeds increase, elite skiers show a distinct “pre-preparation phase” with a more forward pole plant and increased *D*COM-_pole plant_ to gain sufficient time to provoke a pre-activation of muscles before peak pole forces occur (Stöggl and Holmberg 2011; Carlsen et al. 2018). In general, with increased pole length, the distance between pole tip and ankle at pole plant is reduced. This results in a more upright posture during the cycle (less COM_z_), reduced *D*COM-_pole plant_, and causes a smaller external moment arm and torque in the working joints (Carlsen et al. 2018). Such a strategy is probably beneficial for reducing the O_2_-cost and increasing performance at low-to-moderate speeds and moderate-to-steep inclines. However, at higher speeds, skiers must increase their *D*COM-_pole plant_ to gain a sufficient time for pre-activation before peak pole force occurs (Stöggl and Holmberg 2011).

**Fig. 4:**
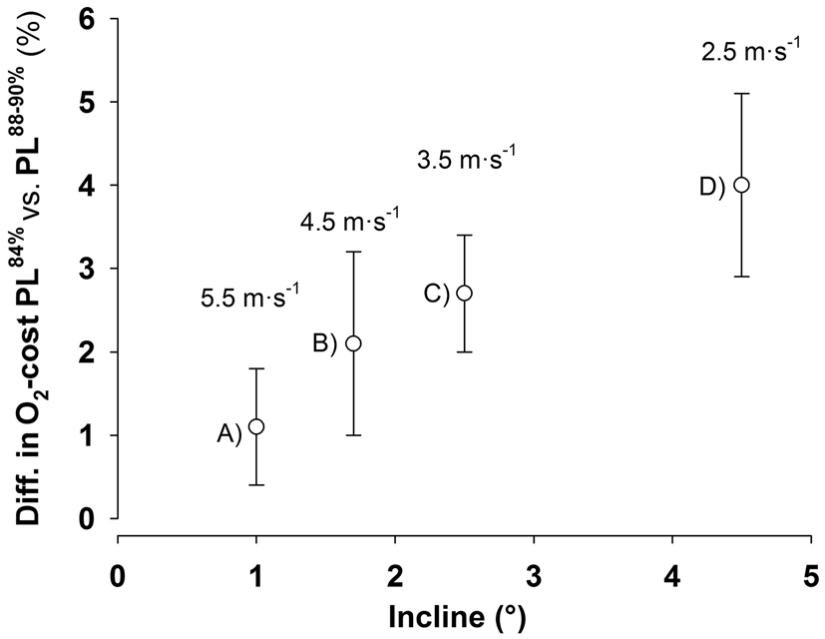
Difference in O_2_-cost (%) between pole lengths of 84% and 88 or 90% of body height as a function of incline and speed. A is data from the present study (*n* = 10), B and D are from Carlsen et al. (2018) (*n* = 13), and C is from Losnegard et al. (2017) (*n* = 9). Data represent mean values and error bars show the 95% CI. All data are from the same lab and rollerskis (including similar friction), but with different subjects participated in the different studies

Second, the displacement of COM_z_ was less for the longer pole lengths at submaximal speeds (4.5–6.0 m s^−1^), which corresponds to the previous findings (Carlsen et al. 2018; Losnegard et al. 2017). Reduced vertical displacement of the COM during the cycle has been shown to be beneficial for reducing the O_2_-cost in DP. This is based on findings that considerable work is done by the lower limbs to extend the body to an upright position during the repositioning phase (Losnegard et al. 2017; Zoppirolli et al. 2015). Indeed, the present study demonstrates that skiers had ~ 1% lower O_2_-cost with PL^90%^ than PL^84%^. Notably, this O_2_-cost difference between pole lengths was lower than in the previous studies (2–4%), supporting the suggestion from Carlsen et al. (2018) that the advantage of longer poles with regard to O_2_-cost and COM_z_ increases with the steepness of the incline with subsequent lower speeds. In fact, Fig. 4 shows an almost perfect correlation between incline or speed and the relative difference in O_2_-cost between self-selected and long poles in recent studies conducted in our lab. Moreover, the typical error (expressed as % coefficient of variation) for rollerski O_2_-cost in our lab is 1.2% (Losnegard et al. 2013), implying that the present findings of 0.6–2.1% between PLs are at the lower end for verifying substantial true effects. In addition, kinematical data collected during the peak velocity test showed no differences in COM_z_ between pole lengths during any test occasions, which strengthens the idea that incline/speed, performance, and COM_z_ are closely related when investigating effects of pole lengths in DP.

### Differences between pole lengths after the training period with long poles

Longer pole lengths induce a different movement pattern during DP than the skiers’ self-selected pole lengths, as shown in the present and previous studies (Carlsen et al. 2018; Losnegard et al. 2017). Thus, it has been hypothesized that skiers would have enhanced their performance even more if they had been allowed to practice for a period with longer poles before testing (Hansen and Losnegard 2010). The present study is the first to investigate how training with longer poles influences peak velocity and O_2_-cost compared to poles of self-selected lengths. Although skiers increased peak velocity and reduced their submaximal O_2_-cost with PL^90%^, the changes were not different to the control condition using PL^84%^. This implies that high-level skiers are able to adjust their technique rapidly, illustrated by the differences between pole lengths in peak velocity at the three timepoints (Fig. 1). Hence, based on the present study, combined with the previous studies (Fig. 4), the effect of pole lengths on O_2_-cost is strongly influenced by the slope and/or the speed. However, it should be noted that even though the skiers had no or little experience with PL^90%^ in DP, their self-selected pole length in ski skating was ~ 90% of body height. As the skating technique V2 (Gear 3) is similar to DP with respect to lowering the COM during the poling phase (Myklebust et al. 2014), prior experience with longer poles in V2 could potentially have influenced the results at Pretest. Nevertheless, no clear differences were seen on changes in O_2_-cost or performance between pole lengths after the training intervention. Moreover, the present study investigated peak velocity during a short-duration time to exhaustion test, where technical ability is a determinative factor for the performance. Our results cannot be directly extrapolated to other tests, such as those, where muscle fatigue is a more important determination of performance, which needs to be further elucidated.

### Practical applications

The effects of pole lengths on O_2_-cost and performance seem highly related to type of terrain and speed. This is strengthen by the findings that the reduced performance with longer poles was not inversed with training, at least over a short training period. Based on the present and previous studies, the clear positive effects of pole lengths of 90% of body height seems evident in the uphill's and low speeds, while pole lengths of 84% of body height are more beneficial in the flat at high speeds. As these factors seems closely related to the displacement of COM_z_, poling time, and external moment arm between poles and COM, the present findings may contribute to enhance our understanding of performance limiting factors in DP. Moreover, the present findings seem highly relevant from a rule perspective. Since performance was reduced with longer poles at high speeds and these segments seems as great importance for the final outcome, the necessity of the pole length restrictions provided by FIS should be reconsidered.

Importantly, the present study was done on a rollerski treadmill, and investigation of outdoors is warranted. The latter is of great importance, since air-drag may potentially influence the results due to the more upright position with longer poles, and thus have a greater negative impact at high skiing speeds. Finally, although the training period did not influence kinematical factors or performance between pole lengths, the training program clearly improved peak velocity and improved technique with the skiers’ self-selected pole lengths in DP. Although these effects could be related to better familiarization of skiing at high speeds on the treadmill, as no control group was included, we argue that variation in training with longer poles could be beneficial for the skiers technique with self-selected pole lengths. This is based on the fact that the increased peak velocity with PL^84%^ was accompanied by less total displacement of COM_z_ and a higher COM at pole plant, and increased poling and reposition time (with lower poling frequency) at high speed. These technical differences are similar to what found when increasing to longer pole lengths in the previous studies (Carlsen et al. 2018; Losnegard et al. 2017). Hence, future studies should investigate if variation with training with longer pole lengths could provide performance enhancement when using pole lengths of 84% of body height.

## Conclusion

Using PL^84%^, skiers were able to reach higher peak velocity compared to PL^90%^, but O_2_-costs and COM_z_ were reduced with PL^90%^ compared to PL^84%^ at moderate speeds. Eight sessions of training with PL^90%^ did not influence the difference between PL^84%^ and PL^90%^ in terms of O_2_-cost, kinematics, or peak velocity.

## Abbreviations

COM: Center of mass
*D*COM: Distance between COM and poles
DP: Double poling
PL: Pole length
PL84%: Pole length of 84% of body height
PL90%: Pole length of 90% of body height
